# Ancestry and TPMT-VNTR Polymorphism: Relationship with Hematological Toxicity in Uruguayan Patients with Acute Lymphoblastic Leukemia

**DOI:** 10.3389/fphar.2020.594262

**Published:** 2020-11-09

**Authors:** Gabriela Burgueño-Rodríguez, Yessika Méndez, Natalia Olano, Agustín Dabezies, Bernardo Bertoni, Jorge Souto, Luis Castillo, Julio da Luz, Ana María Soler

**Affiliations:** ^1^ Laboratorio de Genética Molecular Humana, Centro Universitario Regional (CENUR) Litoral Norte-Sede Salto, Universidad de la República (UdelaR), Salto, Uruguay; ^2^ Servicio Hemato Oncológico Pediátrico (SHOP), Centro Hospitalario Pereira Rossell (CHPR), Montevideo, Uruguay; ^3^ Departamento de Genética, Facultad de Medicina, Universidad de la República, Montevideo, Uruguay

**Keywords:** Acute Lymphoblastic Leukemia, TPMT, TPMT-VNTR, NUDT15, 6-MP, Pharmacogenomics, Ancestry, Hematological toxicity

## Abstract

6-Mercaptopurine (6-MP) is a thiopurine drug widely used in childhood acute lymphoblastic leukemia (ALL) therapy. Genes such as *TPMT* and *NUDT15* have an outstanding role in 6-MP metabolism. Mutations in both genes explain a significant portion of hematological toxicities suffered by ALL Uruguayan pediatric patients. A variable number tandem repeat in the *TPMT* promoter (*TPMT-*VNTR) has been associated with *TPMT* expression. This VNTR has a conservative architecture (AnBmC). To explore new causes of hematological toxicities related to ALL therapy, we genotyped the *TPMT-*VNTR of 130 Uruguayan pediatric patients. Additionally, individual genetic ancestry was estimated by 45 ancestry-informative markers (AIMs). Hematological toxicity was measured as the number of leukopenia events and 6-MP dose along the maintenance phase. As previously reported, we found TPMT*2 and TPMT*3C alleles were associated to *TPMT-*VNTR A2BC and AB2C, respectively. However, contrasting with other reports, TPMT*3A allele was found in a heterogeneous genetic background in linkage equilibrium. Patients carrying more than 5 A repeats present a significant higher number of leukopenia events among patients without *TPMT* and/or *NUDT15* variants. Native American ancestry and the number of A repeats were significantly correlated with the number of leukopenia events. However, the correlation between Native American ancestry and the number of leukopenia events was lost when the number of A repeats was considered as covariate. This suggests that *TPMT-*VNTR alleles are more relevant than Native American ancestry in the hematological toxicity. Our results emphasize that *TPMT*-VNTR may be used as a pharmacogenetic biomarker to predict 6-MP-related hematological toxicity in ALL childhood therapy.

## Introduction

Childhood acute lymphoblastic leukemia (ALL) is the most frequent children cancer worldwide. Uruguay is not an exception. The 5-year disease-free survival (DFS) in this country is similar to that observed in developed countries (Castillos et al., 2012). Although ALL treatments have been improved, approximately 20% of the patients suffer a diversity of adverse side effects due to the nonspecific action and the narrow therapeutic range of the drugs.

In Uruguay, Berlin-Frankfurt-Münster (IC-BFM) protocol is used to treat ALL pediatric patients. In this protocol, patients are classified as standard, intermediate, and high risk according to age of diagnosis, white blood cell (WBC) count, blast number in peripheral blood at day 8, blast percentage in the bone marrow at days 15 and 33, and the presence of translocations. The overall chemotherapy consists in a two-year (104 weeks) treatment encompassing many phases. According to the risk group, the maintenance phase varies between 63 and 74 weeks. In this phase, patients received 50 mg/m^2^/day of 6-mercaptopurine (6-MP) and 20 mg/m^2^/week of methotrexate (MTX). During the maintenance phase, complete blood count (CBC) is performed weekly along the first two months and then every two or three weeks.

Thiopurine S-methyltransferase (TPMT) is a cytosolic enzyme, in which physiological function remains uncertain. TPMT catalyzes the *S*-methylation of thiopurine drugs such as azathioprine, 6-MP, and 6-thioguanine (6-TG), which are widely used in autoimmune, inflammatory, and cancer diseases (Spire-Vayron de la Moureyre et al., 1998; Appell et al., 2013; Chouchana et al., 2014).

Normally, 6-MP and 6-TG are considered as purines and are metabolized to thioguanine nucleotides (TGNs) which will be incorporated into DNA, causing damage and triggering further cellular apoptosis (Pan and Nelson, 1990; Kotur et al., 2012). In the hematopoietic tissue, TPMT has an outstanding role in the downregulation of TGN formation due to its S-methyltransferase activity. There is therefore an inverse relationship between the activity of TPMT and active TGN metabolites’ concentration (Relling et al., 2011).

TPMT illustrates one of the most characteristic examples of pharmacogenetics utility, attempting to a personalized drug therapy. To date, more than 44 *TPMT* mutant alleles have been described (https://www.hmv.liu.se/tpmtalleles), but TPMT*2, TPMT*3A, and TPMT*3C alleles represent more than 95% of inherited TPMT deficiency (Ameyaw et al., 1999; Chrzanowska et al., 2012). Furthermore, within the *TPMT* gene promoter, there is a variable number tandem repeat (VNTR) region (Spire-Vayron de la Moureyre et al., 1998; Spire-Vayron de la Moureyre et al., 1999), whose composition and number of repeats have been associated with *TPMT* expression (Zukic et al., 2010; Kotur et al., 2012; Kotur et al., 2015). This GC-rich VNTR region (*TPMT*-VNTR) has a three-element architecture, two of them being variable in their copy number (AmBnC). According to Vergnaud and Denoeud (2000) criteria, this VNTR is considered a minisatellite, and, hitherto, 19 different alleles have been described (Urbančič et al., 2018).

It has already been reported that, in Uruguayan pediatric patients under ALL therapy, approximately 30% of the hematological toxicities are explained by SNPs at *TPMT* and *NUDT15* genes (Soler et al., 2018). This result demonstrates once again the importance of specific studies for each population, especially in admixed ones such as Uruguayan population. The aim of this work is to analyze the *TPMT*-VNTR in ALL pediatric patients from Uruguay and determine its association with 6-MP hematological toxicity (measured as the number of leukopenia events and 6-MP cumulative dose) in the maintenance phase; taking individual genetic ancestry into account.

## Methodology

### Patients and Clinical Data

DNA samples of 130 ALL Uruguayan pediatric patients aged between 1 and 15 years were analyzed. All patients were followed up at the Servicio Hemato Oncológico Pediátrico—Centro Hospitalario Pereira Rossell, Montevideo, Uruguay, and treated with the IC-BFM protocol (58 according to ALL-IC-BFM 2002 and 72 according to ALL-IC-BFM 2009). The collected clinical data were 6-MP dose and the number of leukopenia events along the first 32 weeks of the maintenance phase. The cumulative 6-MP dose was calculated at weeks 8, 16, 24, and 32. Additionally, we calculated the mean weekly 6-MP dose (mg/m^2^). On the contrary, the hematological toxicity (leukopenia events’ grades 3 and 4) was analyzed as a whole and within eight-week intervals (1–8, 9–16, 17–24, and 25–32 weeks). The data analyzed in this study were collected blinded to genotypes from the patients’ medical files by the two corresponding researchers.

The protocol and procedure employed in this study was approved by the CENUR Litoral Norte, Universidad de la República, institutional ethics committee, and informed consent was obtained from parents, guardians, and/or patients, as required (Exp. N° 311170-000332-17, www.expe.edu.uy).

### *TPMT*, *TPMT*-VNTR, and *NUDT15* Genotyping

Genomic DNA was isolated from peripheral blood leukocytes by the saline extraction method (Miller et al., 1988). Patients were previously genotyped for *TPMT* and *NUDT15* genes which are the most common variants (Soler et al., 2018). The number and type of tandem repeats were determined by *TPMT* first-exon PCR amplification, including the promoter region, followed by Sanger sequencing. Furthermore, in order to discard other possible mutations, the *TPMT* gene was completely characterized by the amplification and sequencing of its nine exons. In addition, since *NUDT15* variants analyzed by Soler et al. (2018) were in exons 1 and 3, we also sequenced *NUDT15* exon 2. Amplification primers and PCR conditions are shown in Supplementary Table S1.

### Linkage Disequilibrium

*TPMT*-VNTR genotypes and *TPMT* genotypic data previously reported by Soler et al. (2018) were used to estimate the gametic phase, applying Arlequin software v3.5.2 (Excoffier and Lischer, 2010). For each haplotype, we estimated the normalized linkage disequilibrium (D’).

### Ancestry

A total of 45 ancestry-informative markers (AIMs) located along all autosomes were genotyped (Supplementary Table S2). These AIMs were selected from the SNP panel published by Yaeger et al. (2008). Nineteen of them were performed by the SNaPshot multiplex system (Thermo Fisher Scientific, Waltham, Massachusetts, United States) and 26 by MassARRAY SNP genotyping (Agena Bioscience Inc., San Diego, United States). In order to estimate the European, Amerindian, and African ancestry, Structure software (Pritchard et al., 2000) was used with 100,000 iterations for the burn-in period and 1,000,000 additional iterations. The parental populations included 42 Europeans (Coriell’s North American panel), 37 West Africans (nonadmixed Africans living in London, United Kingdom, and South Carolina, United States), and 30 Native Americans (15 Mayans and 15 Nahuas), who were genotyped on an Affymetrix 100 K SNP chip (data were kindly provided by Dr. Laura Fejerman (University of California, San Francisco)).

### Statistical Analysis

Allele and genotype frequencies were estimated by gene counting. Correlation between clinical data and the number of repeats (A, B, and A + B) was analyzed by Spearman correlation test. Additionally, patients were classified into two groups according to the number of A repeats (A < 5 and A ≥ 5). The association between these two groups with 6-MP cumulative dose and the number of leukopenia events was analyzed by variance analyses (ANOVA) and Mann–Whitney test, respectively. Furthermore, association between individual ancestry, clinical data, and genetic information was analyzed by ANOVA, Spearman, and partial correlation. Classification tree was built to cluster the patients according to the number of leukopenia events, using the chi-squared automatic interaction detection (CHAID) algorithm. The categorical variables considered were the presence or absence of *TPMT* and/or *NUDT15* variants, A repeats’ groups, and Native American ancestry (cutoff 15%). All analyzes were carried out in the total sample and in the sample subdivided by the presence or absence of *TPMT* and *NUDT15* variants. Statistical analysis was performed in R statistical package with a CI of 95%, with exception of the classification tree which was carried on SPSS 22.0.

## Results

### *TPMT*-VNTR Genotypic and Allelic Frequencies

*TPMT* and *NUDT15* exon sequencing did not reveal any other mutation to those previously reported by Soler et al. (2018). Regarding *TPMT*-VNTR, we identified 18 different genotypes and 10 different alleles. The total number of VNTR repeats (A + B + C) ranged from 6 (ABC/ABC) to 14 (A5BC/A5BC) (Table 1).

**TABLE 1:** *TPMT*-VNTR genotype frequencies and *TPMT* genotype.

*TPMT-*VNTR		*n*	Freq. (%)	*TPMT* (*n*)	Group*
3/3	ABC/ABC	1	0.77	1*/*3A	1
3/4a	ABC/A2BC	1	0.77	*1/*1	1
3/5a	ABC/A2B2C	1	0.77	*1/*1	1
4a/4a	A2BC/A2BC	33	25.38	*1/*1 (29)	1
				*1/*2 (1)	
				*1/*3A (3)	
4a/4b	A2BC/AB2C	4	3.08	*1/*1	1
4a/5a	A2BC/A2B2C	44	33.85	*1/*1 (43)	1
				*1/*3A (1)	
4a/5b	A2BC/A3BC	1	0.77	*1/*1	2
4a/6a	A2BC/A2B3C	11	8.46	*1/*1	1
4a/6b	A2BC/AB4C	2	1.54	*1/*1	1
4a/6c	A2BC/A4BC	2	1.54	*1/*1	2
4a/7a	A2BC/A5BC	3	2.31	*1/*1	2
4b/5a	AB2C/A2B2C	2	1.54	*1/*1 (1)	1
				*1/*3C (1)	
5a/5a	A2B2C/A2B2C	15	11.54	*1/*1 (13)	1
				*1/*3A (2)	
5a/5b	A2B2C/A3BC	1	0.77	*1/*1	2
5a/6a	A2B2C/A2B3C	5	3.85	*1/*1	1
5a/7a	A2B2C/A5BC	2	1.54	*1/*1	2
6b/8a	AB4C/A6BC	1	0.77	*1/*3A	2
7a/7a	A5BC/A5BC	1	0.77	*1/*3A	2
**Total**	—	**130**	—	—	—

Freq.: *TPMT-*VNTR genotype frequency. *1): number of A repeats < 5; 2): number of A repeats ≥ 5.

The most frequent *TPMT*-VNTR genotypes were *4a/*5a (33.85%), *4a/*4a (25.38%), *5a/*5a (11.54%), and *4a/*6a (8.46%). Therefore, *TPMT*-VNTR alleles *4a (51.54%), *5a (32.59%), and *6a (6.15%) explain more than 90% of variability (Table 1 and 2).

**TABLE 2:** Linkage disequilibrium between *TPMT-*VNTR alleles and *TPMT* alleles obtained from estimated haplotype combinations.

*TPMT*-VNTR alleles		Freq. (%)	*TPMT* allele	*n*	D′	*r* ^2^	*X* ^2^	*P*
*3	ABC	1,54	*1	3	-0.217	0.017	4,325	0.038
			*3A	1	0.223	0.022	5,640	0.018
*4a	A2BC	51,54	*1	130	0.118	0.001	0.170	0.680
			*2	1	1,000	0.004	0.944	0.331
			*3A	3	-0.138	0.001	0.188	0.665
*4b	AB2C	2,31	*1	5	-0.130	0.009	2,344	0.126
			*3C	1	1,000	0.163	42,497	**<0.0001**
*5a	A2B2C	32,69	*1	82	0.444	0.004	1,099	0.295
			*3A	3	-0.320	0.002	0.464	0.496
*5b	A3BC	0,77	*1	2	1,000	0.000	0.089	0.765
*6a	A2B3C	6,15	*1	16	1,000	0.003	0.753	0.386
*6b	AB4C	1,15	*1	3	1,000	0.001	0.134	0.714
*6c	A4BC	0,77	*1	2	1,000	0.000	0.089	0.765
*7a	A5BC	2,69	*1	6	-0.105	0.007	1,795	0.180
			*3A	1	0.112	0.010	2,522	0.112
*8a	A6BC	0,38	*3A	1	-1,000	0.087	22,724	**<0.0001**

Freq.: *TPMT-*VNTR allele frequency. D′: normalized linkage disequilibrium coefficient. *r* ^2^: correlation coefficient. *X* ^2^: chi-square. *p* = *p* value.

Six different *TPMT*-VNTR genotypes (*3/*3, *4a/*4a, *4a/*5a, *5a/*5a, *6b/*8a, and *7a/*7a) were found in the nine heterozygous TPMT*3A/*1 patients. The TPMT*2/*1 and TPMT*3C/*1 patients have the *4a/*4a and *4b/*5a *TPMT*-VNTR genotypes, respectively (Table 1).

Estimation of the gametic phase showed the TPMT*3A allele associated to five *TPMT-*VNTR alleles (*3, *4a, *5a, *7a, and *8a), whereas TPMT*2 and TPMT*3C alleles were associated to *TPMT-*VNTR alleles *4a and *4b, respectively. With exception of haplotypes *3-TPMT*1, *3-TPMT*3A, *4a-TPMT*3C, and *8a-TPMT*3A, the remaining haplotypes were found in linkage equilibrium (Table 2).

### Association of *TPMT*-VNTR with Hematological Toxicity

We found a significant correlation between the number of A repeats and the number of leukopenia events in the interval 1–8 (*p* = *0.008*) and when the 32 weeks were analyzed as a whole (*p =* 0.009) (Table 3). Graphical representation of total leukopenia events at week 32 showed a trend toward a greater number of leukopenia events when the number of A repeats was equal or higher than five (Supplementary Figure S1). Therefore, patients were classified into two groups according to the number of A repeats: risk group 1 with less than five A repeats and risk group 2 with five or more A repeats. When the number of leukopenia events was compared among the risk groups by the Mann–Whitney test, the number of leukopenia events was significantly greater in risk group 2 than that in risk group 1 at intervals 1–8 and 17–24 and considering the 32 weeks as a whole (Table 3).

**TABLE 3:** Association between the number of leukopenia events and 1) the number of A, B, and A + B repeats and 2) risk groups.

Interval (weeks)		Spearman correlation (1)						Mann–Whitney (2)				
		Number of A repeats		Number of B repeats		A + B repeats		Number of leukopenia events				*p*
		*R*	*p*	*R*	*p*	*r*	*P*	Risk group 1		Risk group 2		
								*N*	Mean	*N*	Mean	
1–8	Total	0.245	***0.008***	-0.122	0.116	-0.007	0.474	88	0.61	8	1.63	***0.010***
	*wt*							72	0.54	6	1.50	***0.044***
	mut							16	0.94	2	2	0.105
9–16	Total	0.112	0.140	0.056	0.294	0.106	0.151	88	0.85	8	1.62	0.105
	*wt*							72	0.71	6	1.17	0.328
	mut							16	1.50	2	3	0.163
17–24	Total	0.166	0.054	-0.170	0.050	-0.070	0.250	87	0.48	8	1.25	**0.025**
	*wt*							71	0.41	6	1.17	0.068
	mut							16	0.81	2	1.50	0.216
25–32	Total	0.137	0.093	0.099	0.172	0.126	0.114	86	0.34	8	0.63	0.154
	*wt*							71	0.28	6	0.67	0.148
	mut							15	0.60	2	0.50	0.669
Total	Total	0.240	***0.009***	-0.075	0.233	0.044	0.336	88	2.27	8	5.13	***0.002***
(32 weeks)	*wt*							72	1.93	6	4.50	***0.012***
	mut							16	3.81	2	7	0.078

1) Association between the number of leukopenia events and the number of A, B, and A + B repeats was done by Spearman test. 2) The number of leukopenia events between risk groups was compared by Mann–Whitney test. Total: total sample; *wt:* patients without TPMT or NUDT15 variants; mut: patients with TPMT and/or NUDT15 variants. *r*: Spearman correlation coefficient. *p*: *p* value. *N*: number of individuals. Risk group 1: A < 5. Risk group 2: A ≥ 5. Mean: mean number of leukopenia events’ grade 3 or 4.

As the number of leukopenia events is strongly associated with *TPMT* and *NUDT15* variants (Relling et al., 2011; Cargnin et al., 2018; Soler et al., 2018), we also subdivided the sample according to the presence or absence of *TPMT* and/or *NUDT15* variants. The association between risk groups and the number of leukopenia events remains significant only in the subsample without *TPMT* and/or *NUDT15* variants. This association was observed in the interval 1–8 and in the total number of leukopenia events at week 32 (Table 3).

We did not detect significant correlations between 6-MP cumulative dose and the *TPMT*-VNTR length (A + B) as well as with the number of A or B repeats separately (Supplementary Table S3). Moreover, 6-MP cumulative dose did not show an association with risk groups by ANOVA neither in the total sample nor in the sample subdivided by the presence and absence of *TPMT* and/or *NUDT15* variants (data not shown).

### Ancestry, Hematological Toxicity, and *TPMT*-VNTR

Ancestry was determined in 111 of 130 patients. European, Native American, and African ancestral proportions were 73.9 ± 14.8%, 16.9 ± 11.7%, and 9.12 ± 7.4% (mean ± SD), respectively (Supplementary Figure S2).

**FIGURE 1:**
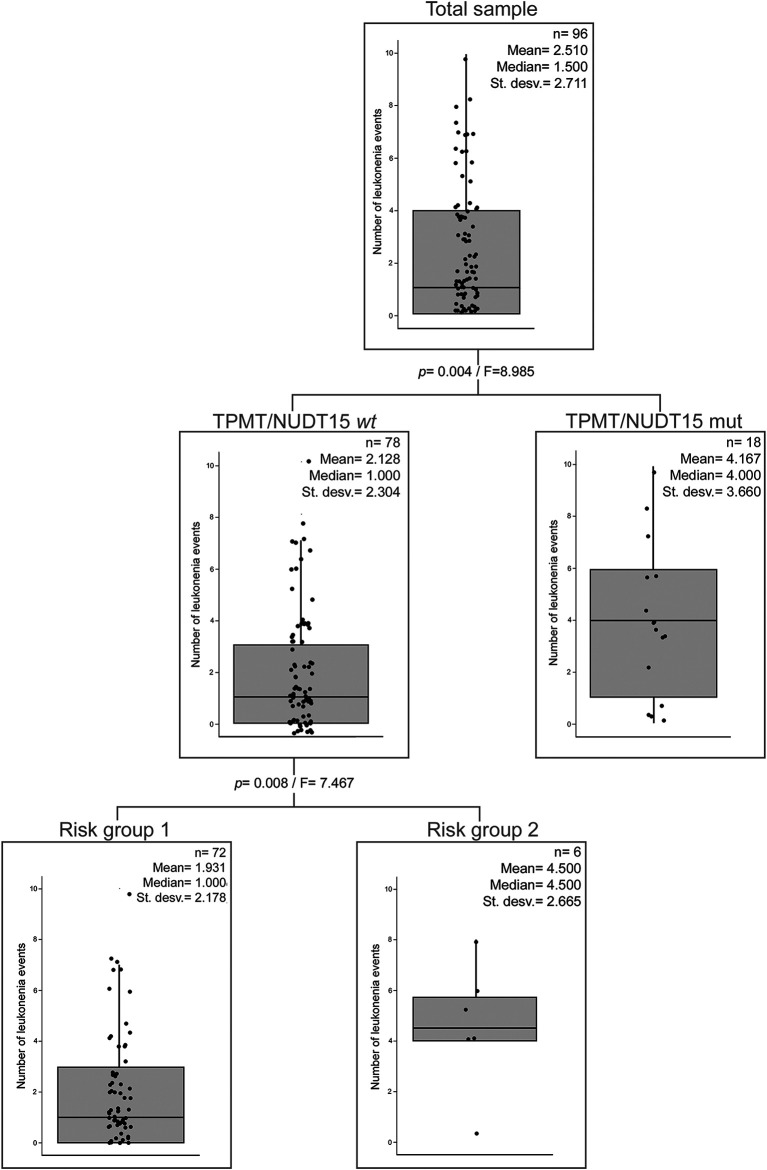
Classification tree of the number of leukopenia events. Presence/absence of *TPMT* and/or *NUDT15* mutations, risk groups, and Native American ancestry (cutoff = 15%) were taken as categorical variables. *TPMT/*NUDT15w*t*: TPMT*1 and NUDT15*1. TPMT/NUDT15mut: *TPMT* and/or *NUDT15* mutated. Risk group 1: A < 5. Risk group 2: A ≥ 5.

There is a significant negative correlation between the number of leukopenia events and Native American ancestry in patients without *TPMT* and/or *NUDT15* variants (Supplementary Table S4). Moreover, risk group 2 patients showed a significantly higher European and lower Native American ancestry than patients from risk group 1 among patients without *TPMT* and/or *NUDT15* variants (Supplementary Figures 2C,D). In order to analyze the relevance of the abovementioned variables, we additionally performed a partial correlation analysis between the number of leukopenia events and patients’ Native American ancestry using risk groups as covariate. This analysis did not show a significant correlation (*p* = 0.120). Furthermore, classification tree first divided the sample by the presence or absence of *TPMT* and/or *NUDT15* variants and then by risk groups resulting in three groups (two nodes). Native American ancestry was not a significant variable to explain the number of leukopenia events (Figure 1).

6-MP cumulative dose does not show a significant correlation with European, Native American, or African ancestry in the total sample.

## Discussion

As it has been widely reported, *TPMT* and *NUDT15* genes have an outstanding role in the 6-MP metabolic pathway. Variants in both genes induce loss of their enzymatic activity and the subsequent accumulation of TGNs, resulting in the presence of adverse drug reaction due to 6-MP (Fabre et al., 2004; Yang et al., 2014; Moriyama et al., 2016; Soler et al., 2018; Cao et al., 2020). However, patients without *TPMT* and/or *NUDT15* variants also suffer 6-MP adverse effects. A VNTR region in the *TPMT* promoter has been postulated to modulate TPMT activity (Spire-Vayron de la Moureyre et al., 1998) and hence may explain part of the hematological toxicity due to 6-MP.

In our study population, an admixed one, overall *TPMT-*VNTR allele frequencies did not differ significantly from the previously reported for Portuguese and Mozambique (Alves et al., 2002), Asian British (Marinaki et al., 2003), and Serbian populations (Zukic et al., 2010; Kotur et al., 2012). The most frequent alleles found were *4a and *5a followed by *6a, which were similar to those observed in other populations (Alves et al., 2002; Marinaki et al., 2003; Zukic et al., 2010; Urbančič et al., 2018). Interestingly, alleles *3 and *4b were found at higher frequencies than those observed in European populations and similar to those reported for Mozambique and Asian British populations, respectively (Alves et al., 2002; Marinaki et al., 2003; Urbančič et al., 2018). This fact could be explained by the contribution of Native American and African populations to the Uruguayan genetic pool (Sans et al., 1997; Buchelli and Cabella., 2010; Bonilla et al., 2015).

Genotypic and linkage disequilibrium (LD) analyses showed that, with exception to the *8a allele, the functional TPMT*1 allele is linked to the rest of the *TPMT*-VNTR alleles. In concordance with the previous reports, TPMT*2 and TPMT*3C alleles were found linked to *TPMT*-VNTR *4a and *4b alleles, respectively (Alves et al., 2001; Marinaki et al., 2003; Urbančič et al., 2018).

On the contrary, TPMT*3A (the most common deficient allele) was linked to six different *TPMT*-VNTR alleles. Six out of nine TPMT*3A alleles were linked in equilibrium to the most frequent *TPMT*-VNTR alleles (*4a and *5a), similar to what Spire-Vayron de la Moureyre et al. (1998) reported for a European population. Moreover, the *7a allele (the fourth most frequent *TPMT*-VNTR allele) was also found in linkage equilibrium with the TPMT*3A allele. Different to previous studies, we did not observe linkage between TPMT*3A and *6b (Alves et al., 2001; Marinaki et al., 2003). Additionally, TPMT*3A allele was in LD only with *TPMT*-VNTR alleles found in low frequencies (*3 and *8a). This result contrasts with previous studies, where TPMT*3A was found in LD with *5a, *6a, and ABnC (n > 2) alleles (Yan et al., 2000; Alves et al., 2001; Marinaki et al., 2003; Urbančič et al., 2018) (Table 2).

The linkage between *TPMT-*VNTR with the variable number of A repeats and TPMT*3A allele, added to the absence of LD when all alleles were considered (*p =* 0.198), does not adjust to the evolutionary model proposed by Urbančič et al. (2018). These differences may be explained by several reasons: 1) the loss of LD by recombination between the TPMT*3A allele and the common *TPMT*-VNTR alleles as suggested by Marinaki et al. (2003); 2) the heterogeneity in the Caucasic population that contributes to the Uruguayan genetic pool; and 3) microevolutionary events as genetic drift or founder effects occurring in the Uruguayan population.

Several studies had reported that *TPMT*-VNTR modulates the expression of the *TPMT* gene, possibly at the transcriptional level. Some authors had reported an inverse correlation between the overall repeat number of *TPMT*-VNTR and TPMT activity (Spire-Vayron de la Moureyre et al., 1998; Yan et al., 2000; Fabre et al., 2004), whereas other authors found a correlation between the number of A or B repeats with TPMT activity and/or with hematological toxicities (Alves et al., 2001; Zukic et al., 2010; Kotur et al., 2012; Kotur et al., 2015). Most of these studies had analyzed the relationship between *TPMT*-VNTR alleles and *TPMT* expression/activity at the molecular or biochemical level. In our study, we focus on the leukopenia events and on 6-MP dose, two indirect clinical measures of TPMT expression/activity.

We found a negative correlation between the number of A repeats and the number of leukopenia events (Table 3). These results agree with *TPMT* gene expression data previously reported by Zukic et al. (2010) and Kotur et al. (2012), Kotur et al. (2015). Interestingly, we did not find any significant correlation between either the total overall repeat number or the number of B repeats with the two clinical measures analyzed, as described by Spire-Vayron de la Moureyre et al. (1998) and Alves et al. (2001). Other study, with kidney transplant recipients receiving azathioprine, reported that patients with *TPMT*-VNTR genotypes composed of more than ten repeats showed a significant reduction in azathioprine dose compared to those with ten or less repeats (Fabre et al., 2004). Although this study does not discriminate between the number of A and B repeats, those individuals (>10 repeats) must have at least one *TPMT*-VNTR allele carrying more than six repeats. These alleles may show a bias toward a higher number of A than B repeats according to reported *TPMT*-VNTR allele frequencies (Urbančič et al., 2018).

According to Kotur et al. (2015), carriers of the *7a allele had the lowest average of *TPMT* gene expression level and the least increase in its expression level during chemotherapy. Interestingly, six of our 11 patients, belonging to risk group 2 (A ≥ 5), carry the *7a allele. Unfortunately, there are no other studies about *TPMT* expression in patients carrying the other *TPMT*-VNTR alleles belonging to risk group 2 (*5b, *6c, and *8a).

The relevance of genetic ancestry in 6-MP therapy has been demonstrated by several examples such as hematological toxicity observed in patients carrying NUDT15 variants, which is more frequent in Asian and South American populations (Yang et al., 2014; Soler et al., 2018). Global ancestry proportions were similar to those observed in previous studies and confirmed the trihybrid structure of the Uruguayan population (Sans et al., 1997; Sans et al., 2006; Bonilla et al., 2015). At the individual level, the observed wide variation was also similar to the previous report (Bonilla et al., 2015) (Supplementary Figure S2A).

Even though we found a significant negative correlation between the total number of leukopenia events and Native American ancestry in patients without *TPMT* and/or *NUDT15* variants (Supplementary Table S4), this correlation was no longer significant when risk groups were taken into consideration (*p* = 0.120). This may show that *TPMT-*VNTR alleles are more relevant than Native American ancestry. A heterogeneous distribution of *TPMT-*VNTR alleles among ancestral populations may explain the loss of the correlation between Native American ancestry and the total number of leukopenia events. However, although *TPMT-*VNTR frequencies in Asian and Native American populations are scarce, these data do not support this hypothesis (Marinaki et al., 2003; Urbančič et al., 2018). Another possible explanation is microevolutionary factors as genetic drift or founder effects, causing different levels of ancestry between risk groups.

Despite the significant association between the number of leukopenia events and risk group 2, as visualized at the classification tree and consistent with previous reports (Zukic et al., 2010; Kotur et al., 2012; Kotur et al., 2015), we must be cautious with this result due to the small sample size and the low number of patients with five or more A repeats analyzed in this study. Nevertheless, considering that Uruguay has only 3.5 million inhabitants and presents approximately 25 new ALL pediatric patients per year, a 130-individual sample represents more than 5 years of ALL patients in our country.

Furthermore, additionally to *NUDT15* and *TPMT*, there are other genes involved in 6-MP metabolism, like *ITPA* or *ABCC4,* which may influence in the hematological toxicity (Hareedy et al., 2015; Tanaka et al., 2015; Tanaka et al., 2018).

Although we found a clear association between risk group 2 and the number of leukopenia events, this association was not reflected in 6-MP dose. This could be due to a weaker effect of *TPMT-*VNTR compared with other genetic factors such as *TPMT* and *NUDT15* variants. Additionally, the decision to modify 6-MP dose is based on empirical information, and thus relative to the patient general state and to medical staff.

To our knowledge, this is the first report that studied the relationship between *TPMT*-VNTR alleles and clinical data, stratifying the sample by *TPMT* and/or *NUDT15* mutations.

In summary, we confirm the previous reported association between *4a and *4b *TPMT-*VNTR alleles with TPMT*2 and TPMT*3C, respectively. However, we did not find the reported association between *TPMT-*VNTR pattern ABnC (n ≥ 2) and TPMT*3A, suggesting a greater heterogeneity in the genetic background of patients carrying this variant. Moreover, a higher number of A repeats might be a risk factor for suffering leukopenia events. Finally, our results support the hypothesis that transcriptional genetic modifiers, such as the VNTR region in the *TPMT* promoter, may be used as a pharmacogenetic biomarker and could contribute to the design of personalized ALL childhood therapy.

## Data Availability Statement

The raw data supporting the conclusion of this article will be made available by the authors, without undue reservation.

## Ethics Statement

The studies involving human participants were reviewed and approved by Comité de Ética en Investigación Institucional, Centro Universitario Regional (CENUR) Litoral Norte, Universidad de la República (UdelaR). Written informed consent to participate in this study was provided by the participants’ legal guardian/next of kin.

## Author Contributions

AS, JL, and GB-R designed the study, analyzed clinical and genetic data, and drafted the manuscript. BB and JS participated in the ancestry analyses. LC, AD, YM, and NO were responsible for patients’ recruitment and helped in the analyses of clinical data.

## Funding

Financial support from Centro Universitario Regional (CENUR) Litoral Norte, Universidad de la República (UdelaR) (2017-2019), and Comisión Sectorial de Investigación Científica-Vinculación Universidad Sociedad y Producción Modalidad 2, Universidad de la República (CSIC-VUSP-Mod. 2 2016, UdelaR).

## Conflict of Interest

The authors declare that the research was conducted in the absence of any commercial or financial relationship that could be construed as a potential conflict of interest.
