# Supplementary material for: Ancestry and TPMT-VNTR Polymorphism: Relationship with Hematological Toxicity in Uruguayan Patients with Acute Lymphoblastic Leukemia
Source: Front Pharmacol. 2020 Nov 9;11:594262. doi: 10.3389/fphar.2020.594262 (PMC7789872; doi:10.3389/fphar.2020.594262)
Supplement: Supplementary file 1 [file DataSheet1_v1.PDF]

Supplementary Table 1. PCR amplification conditions

Gene	Exon	Primer Sequence (5'-3')	Amplicon size (bp)	Denaturalization (temp./time)	Annealing (temp./time)	Extension (temp./time)	
TPMT	1	Fw- GGAGTCTGTGCAACGAGGTA<br>Rv- TGCTAGGCTGTCTACGCTTG	368	94°C/30sec	58°C/45sec	72°C/1min	*
	2	Fw- CAGTGAGCCAAGATCACACTAC<br>Rv- ACTACATCATGCCACAGATGC	544	94°C/30sec	61°C/45sec	72°C/45sec.	
	3	Fw- AGGTTGTTGGGAATATTAAGTGAGA<br>Rv- CCCAAAGAATTCATCATTAAGGCA	239	94°C/30sec	53°C/45sec	72°C/1min	
	4	Fw- CTGCTTTCCTGCATGTTCTTTG<br>Rv- ACAGTGAATCTGCGTGCTAAA	403	94°C/30sec	53°C/45sec	72°C/1min	
	5	Fw- GGC CCT CTT TCC TTG ACT ATT<br>Rv- GGATGTTACACAGGAGGAAGAG	235	94°C/30sec	59°C/45sec	72°C/1min	
	6	Fw- AGGCAGCTAGGGAAAAAGAAAGGT<br>Rv- CAAGCCTTATAGCCTTACACCCAG	694	94°C/30sec	58°C/45sec	72°C/1min	(1)
	7	Fw- AAGTAACTTCTGGCTTCCTTCC<br>Rv- TGCAGTATGCTTCCTATGAGATAAA	295	94°C/30sec	58°C/45sec	72°C/1min	
	8	Fw- GAAGAACATGCCACATCATCAC<br>Rv- CCTCCCAAAGTGCTGGAAATA	253	94°C/30sec	58°C/45sec	72°C/30sec.	
	9	Fw- GAGACAGAGTTTCACCATCTTGG<br>Rv- CAGGCTTTAGCATAATTTTCAATTCCTC	373	94°C/30sec	53°C/45sec	72°C/1min	(1)
NUDT15	2	Fw- CGGCCTTCCAAAAGATTACA<br>Rv- TGATCTAATCACCTCCCAAGG	650	95°C/30sec	58°C/30sec	72°C/45sec.	(2)

All the PCRs were performed using 30 amplification cycles. * DMSO required. (1) Ameway et al., 1999. (2) Moriyama et al., 2016.

Supplementary Table 2. Table of 45 ancestry informative markers (AIMs)

		Allele frequencies			Delta values		
AIMs	Chromosome	African (AF)	European (EU)	Native American (NA)	AF-EU	AF-NA	EU-NA
rs1934393	1	0.222	0.842	0.300	0.620	0.078	0.542
rs2817611	1	0.278	0.952	0.967	0.675	0.689	0.014
rs6684063	1	0.222	0.833	0.167	0.611	0.056	0.667
rs1036543	2	0.750	0.024	0.767	0.726	0.017	0.743
rs842634	2	0.972	0.738	0.233	0.234	0.739	0.505
rs1470524	2	0.222	0.786	0.533	0.563	0.311	0.252
rs10510791	3	0.972	0.488	0.133	0.484	0.839	0.354
rs9310888	3	0.667	0.075	0.000	0.592	0.667	0.075
rs10519979	4	0.167	0.451	0.967	0.285	0.800	0.515
rs1398829	4	0.222	0.976	1.000	0.754	0.778	0.024
rs10515535	5	1.000	0.286	0.133	0.714	0.867	0.153
rs257748	5	0.806	0.381	0.967	0.425	0.161	0.586
rs10484578	6	0.944	0.375	0.067	0.569	0.878	0.308
rs6911727	6	0.139	0.476	1.000	0.337	0.861	0.524
rs9320808	6	0.861	0.095	0.967	0.766	0.106	0.871
rs10248051	7	0.083	0.833	0.200	0.750	0.117	0.633
rs10214949	7	0.281	0.845	0.967	0.564	0.685	0.121
rs10486576	7	0.944	0.900	0.200	0.044	0.744	0.700
rs1898280	8	0.889	0.238	0.833	0.651	0.056	0.595
rs4733652	8	0.917	0.762	0.100	0.155	0.817	0.662
rs10491654	9	0.500	0.286	0.900	0.214	0.400	0.614
rs4013967	9	0.059	0.655	1.000	0.596	0.941	0.345
rs10508349	10	0.056	0.012	0.767	0.044	0.711	0.755
rs1397618	10	0.294	0.952	1.000	0.658	0.706	0.048
rs10501474	11	0.056	0.643	0.800	0.587	0.744	0.157
rs948360	11	0.250	0.974	1.000	0.724	0.750	0.026
rs249847	12	0.056	0.463	0.967	0.408	0.911	0.504
rs4762106	12	0.806	0.095	0.667	0.710	0.139	0.571
rs10507688	13	0.000	0.167	0.733	0.167	0.733	0.567
rs2585901	13	0.765	0.854	0.067	0.089	0.698	0.787
rs10131076	14	0.694	0.071	0.000	0.623	0.694	0.071
rs1451928	14	0.861	0.857	0.200	0.004	0.661	0.657
rs10520678	15	0.154	0.732	1.000	0.578	0.846	0.268
rs9302185	15	0.875	0.190	0.033	0.685	0.842	0.157
rs1004704	16	0.028	0.214	0.867	0.187	0.839	0.652
rs10500505	16	0.056	0.214	0.800	0.159	0.744	0.586
rs10491097	17	0.056	0.647	0.133	0.592	0.078	0.514
rs2253624	17	0.176	1.000	1.000	0.724	0.724	0.000
rs1013459	18	0.233	0.900	0.967	0.667	0.733	0.067
rs12993952	18	0.139	0.927	0.967	0.788	0.828	0.040
rs888861	19	0.000	0.738	0.900	0.738	0.900	0.162
rs708915	20	0.735	0.100	0.733	0.635	0.002	0.633
rs2208139	20	0.833	0.667	0.033	0.167	0.800	0.633
rs2829454	21	0.056	0.274	0.933	0.218	0.878	0.660
rs138022	22	0.833	0.262	0.033	0.571	0.800	0.229

Data obtained from Yaeger et al. (2008).

Supplementary Table 3. Spearman correlation between 6-MP cumulative dose and the number of A, B and A + B repeats.

	A		B		A + B	
	r	*p*	r	*p*	r	*p*
Week 8	-0.077	0.227	0.010	0.461	-0.045	0.333
Week 16	0.051	0.312	-0.040	0.348	-0.025	0.405
Week 24	0.026	0.402	-0.029	0.390	-0.023	0.411
Week 32	0.010	0.461	-0.043	0.341	-0.040	0.352
Mean weekly dose	0.064	0.265	-0,044	0.335	-0.010	0.460

r: Spearman correlation coefficient. *p*: *p*-value

Supplementary Table 4. Spearman correlation between number leukopenia events and individual ancestry.

Interval (weeks)		European		Native American		African	
		r	*p*	r	*p*	r	*p*
1-8	Total	-0.045	0.665	-0.004	0.967	0.053	0.615
	wt	-0.011	0.922	-0.071	0.540	0.053	0.648
	mut	-0.105	0.689	0.211	0.417	0.155	0.551
9-16	Total	0.092	0.375	-0.134	0.199	-0.035	0.737
	wt	0.100	0.388	-0.192	0.095	0.034	0.770
	mut	-0.087	0.739	0.121	0.644	-0.079	0.763
17-24	Total	0.160	0.124	-0.170	0.102	-0.037	0.721
	wt	0.167	0.147	-0.219	0.056	0.016	0.889
	mut	0.130	0.619	-0.070	0.790	-0.179	0.491
25-32	Total	0.094	0.367	-0.046	0.660	-0.060	0.566
	wt	0.090	0.436	-0.069	0.549	-0.038	0.740
	mut	0.165	0.527	-0.032	0.902	-0.102	0.698
Total	Total	0.149	0.153	-0.187	0.070	-0.029	0.780
(32 weeks)	*wt*	0.154	0.181	-0.243	***0.032***	0.011	0.927
	mut	0.038	0.884	0.032	0.902	-0.028	0.915

Total: all patients*; wt:* Patients without *TPMT* or *NUDT15* variants; mut: Patients with *TPMT* and/or *NUDT15* variants. r: Spearman correlation coefficient. *p*: *p*-value.
