# Supplementary material for: Ancestry and TPMT-VNTR Polymorphism: Relationship with Hematological Toxicity in Uruguayan Patients with Acute Lymphoblastic Leukemia
Source: Front Pharmacol. 2020 Nov 9;11:594262. doi: 10.3389/fphar.2020.594262 (PMC7789872; doi:10.3389/fphar.2020.594262)
Supplement: Supplementary file 2 [file DataSheet2_v1.PDF]

Supplementary Figure 1. Number of leukopenia events at week 32 vs the amount of A repeats within the *TPMT*-VNTR.

Supplementary Figure 2. **Ancestry and risk groups**. **A)** Ancestral genetics proportions of 111 patients with ALL. Each vertical bar along the x axis represents an individual and the bars are colored according to the individual genetic ancestry (dark purple: African, violet: European and pink: Native American). **B, C** and **D)** ANOVA of ancestral component (**B**: African European, **C**: and **D**: Native American) for each risk group in *TPMT* and/or *NUDT15* mutated (Mut) and not mutated (*Wt*) patients.
