# Supplementary figures and images for: Ancestry and TPMT-VNTR Polymorphism: Relationship with Hematological Toxicity in Uruguayan Patients with Acute Lymphoblastic Leukemia
Source: Front Pharmacol. 2020 Nov 9;11:594262. doi: 10.3389/fphar.2020.594262 (PMC7789872; doi:10.3389/fphar.2020.594262)

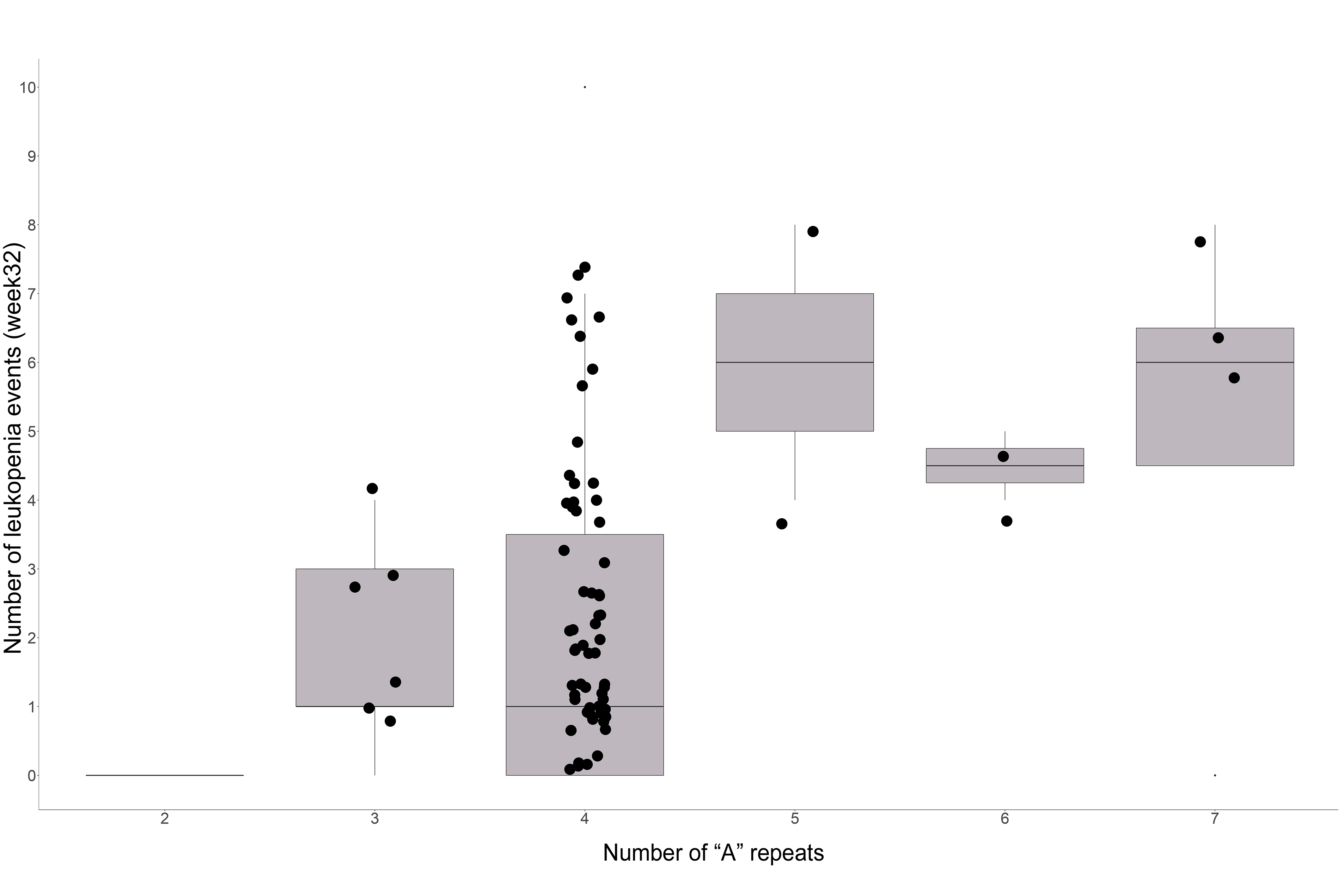

Supplement: Supplementary file 3 [file Image1_v1.JPEG]

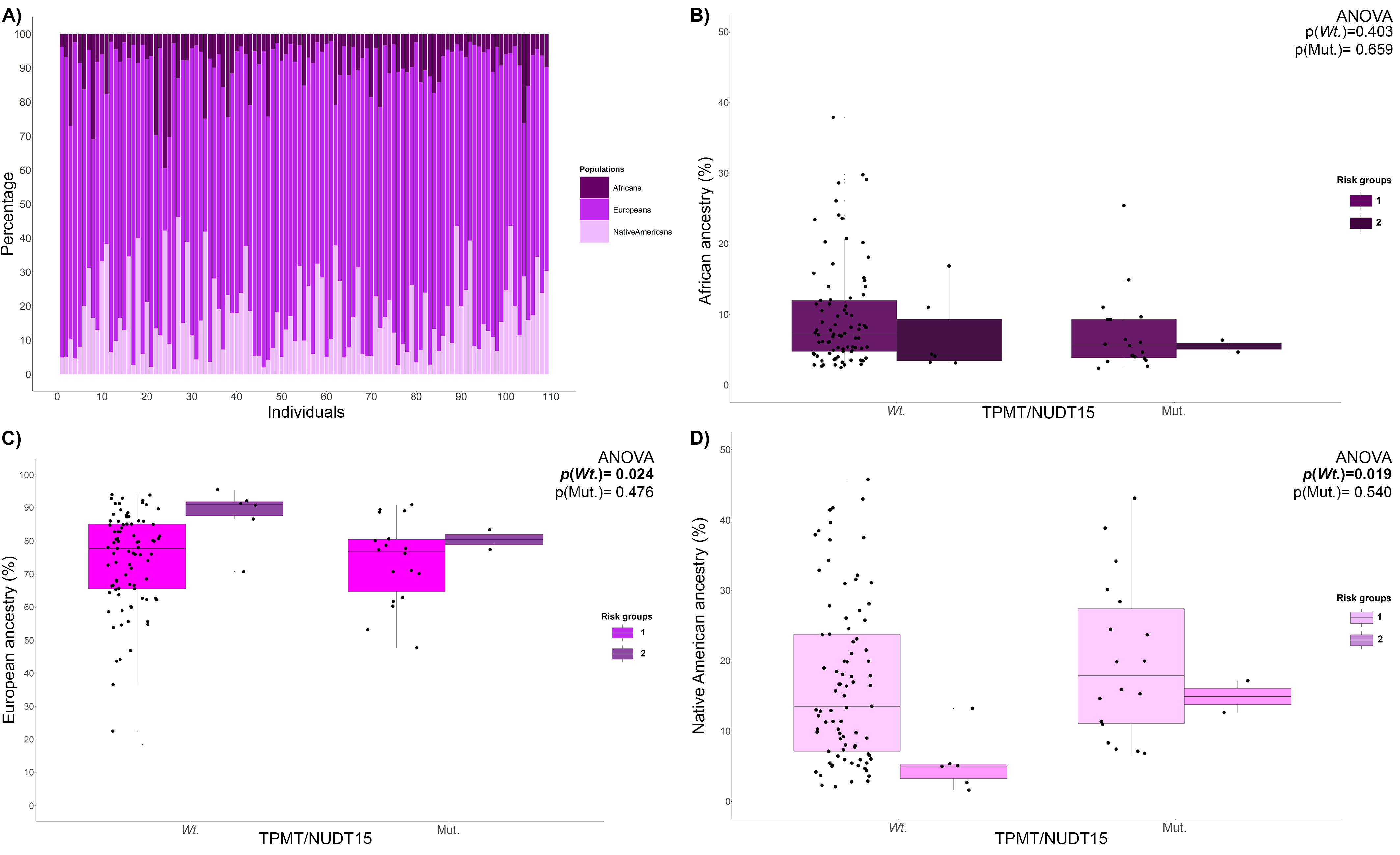

Supplement: Supplementary file 4 [file Image2_v1.JPEG]
